# Integrative genome-wide chromatin signature analysis using finite mixture models

**DOI:** 10.1186/1471-2164-13-S6-S3

**Published:** 2012-10-26

**Authors:** Cenny Taslim, Shili Lin, Kun Huang, Tim Hui-Ming Huang

**Affiliations:** 1 Department of Statistics, The Ohio State University, Columbus, Ohio 43210, USA; 2 Department of Biomedical Informatics, The Ohio State University, Columbus, Ohio 43210, USA; 3 Department of Molecular Medicine/Institute of Biotechnology and Cancer Therapy and Research Center University of Texas Health Science Center, San Antonio, Texas 78229, USA

## Abstract

Regulation of gene expression has been shown to involve not only the binding of transcription factor at target gene promoters but also the characterization of histone around which DNA is wrapped around. Some histone modification, for example di-methylated histone H3 at lysine 4 (H3K4me2), has been shown to bind to promoters and activate target genes. However, no clear pattern has been shown to predict human promoters. This paper proposed a novel quantitative approach to characterize patterns of promoter regions and predict novel and alternative promoters. We utilized high-throughput data generated using chromatin immunoprecipitation methods followed by massively parallel sequencing (ChIP-seq) technology on RNA Polymerase II (Pol-II) and H3K4me2. Common patterns of promoter regions are modeled using a mixture model involving double-exponential and uniform distributions. The fitted model obtained were then used to search for regions displaying similar patterns over the entire genome to find novel and alternative promoters. Regions with high correlations with the common patterns are identified as putative novel promoters. We used this proposed algorithm, RNA-seq data and several transcripts databases to find alternative promoters in MCF7 (normal breast cancer) cell line. We found 7,235 high-confidence regions that display the identified promoter patterns. Of these, 4,167 regions (58%) can be mapped to RefSeq regions. 2,444 regions are in a gene body or overlap with transcripts (non-coding RNAs, ESTs, and transcripts that are predicted by RNA-seq data). Some of these maybe potential alternative promoters. We also found 193 regions that map to enhancer regions (represented by androgen and estrogen receptor binding sites) and other regulatory regions such as CTCF (CCCTC binding factor) and CpG island. Around 5% (431 regions) of these correlated regions do not overlap with any transcripts or regulatory regions suggesting that these might be potential new promoters or markers for other annotation which are currently undiscovered.

## Background

Multicellular organism consists of hundreds of different cell types. A cell typically expresses only a fraction of its genes. Each type of cells become different from others because they activate different sets of genes whose activities turn on and off various biological processes. The process in which a cell determines which genes it will express and when is called *gene regulation*. Because of the multitude of cell types, the regulation of gene expression in complex genomes, such as the human genome, is known to be an extremely complicated process. It is now well accepted that apart from sequence polymorphism and variations, gene regulation in human plays an important role in many disease onset and progression. By matching the gene expression profiles to those of known tumors, researchers can type cancer cells of unknown tissue origin. As such, understanding the mechanism governing regulation of genes is very crucial. For many genes, their expression levels are controlled by attachment of specific proteins known as transcription factors to locations on the DNA to activate or suppress expression of the target genes. The location where transcription factor binds to is known as *promoter region*. Recent discoveries show that regulation of gene expression not only involve the binding of transcription factors in target gene promoters but it also depends on the characterization of the epigenetic events such as histone marks around which DNA is wrapped around [1-3]. Certain histone modification, for example di-methylated histone H3 at lysine 4 (H3K4me2) has been suggested to relax the nucleosome packing, allowing nuclear factors to bind into promoter region and activate gene [1]. Specific chromatin signatures were also reported to be present at gene promoters [4]. Thus, characterization of histone modifications at promoter regions fundamentally contributes toward deciphering of gene expression mechanism. To complicate the process even further, more than half of the human genes has been known to have multiple promoters. Genes that display complex transcription regulation in different cellular conditions or developmental stages have been shown to utilize alternative promoters [5]. Therefore, predicting all these gene promoters including their alternatives are deemed to be important in understanding gene regulation mechanism.

With the rapid availability of high-throughput technologies such as chromatin immunoprecipitation followed by next-generation sequencing (ChIP-seq), scientists can now observe the binding patterns of the protein of interest in the entire genome. Genome-wide identification of promoter is commonly done using antibody against RNA polymerase II (enzyme that are required for gene transcription) [6]. However, due to non-specific binding of Pol-II over the genome and the specific characteristics of antibody against Pol-II, it is hard to predict promoters based on Pol-II enrichment alone. The dynamics of transcribing Pol-II throughout the gene body also makes it hard to pinpoint the exact promoter region. Furthermore, there has been evidence showing that although Pol-II may accumulate at a promoter, the gene is not transcribed. A phenomenon known as RNA Pol-II stalling, which has been shown to occur in Drosophila [7], may also happen in human.

Thus, development of a better promoter identification algorithm is needed to account for these different situations. It is conceivable that promoter regions display unique combination of chromatin and Pol-II patterns. Condition such as Pol-II stalling may display different patterns than those of transcribing genes. As an attempt to address this problem, in this article, we propose a computational method using a finite mixture model to identify promoter signature profiles based on both Pol-II and H3K4me2 binding patterns. We choose to use H3K4me2 pattern because H3K4 di-methylation has been shown to promote transcriptional activities of genes [1]. We scan the genome to find regions which display the identified promoter signatures using the fitted model. We call these regions putative promoters. Aided by RNA-seq data combined with several transcripts databases, we annotate these putative promoters as predicted alternative and novel promoters. We have also found similar patterns exist in regions that have been associated with gene regulatory sites besides promoters such as ER/AR (Estrogen and Androgen Receptor) binding sites. These two proteins have been known to bind to non-promoter regions known as enhancers [8,9]. We have also found genomic regions displaying these Pol-II and H3K4me2 patterns that mapped exclusively to other regulatory regions such as CTCF (CCCTC binding factor) and CpG island.

## Methods

### Data sets and genome annotations

Two ChIP-seq data sets are used to identify patterns of promoters, RNA Pol-II ChIP-seq data and H3K4me2 ChIP-seq data, both from MCF7 (normal breast cancer cell line). RNA-seq (RNA sequencing) data also from MCF7 are used to identify transcripts in the breast cancer cell line including alternative splicing. Genome annotation databases such as non-coding RNA (ie. snoRNA and miRNA), ESTs (Expressed Sequence Tags), CpG island and CTCF (CCCTC binding factor) tracks are downloaded from UCSC genome browser. ER/AR (Estrogen and Androgen Receptor) binding sites are retrieved from HRTBLDb (Hormone Receptor Target Binding Loci) database [10].

### Methods

**Figure 1:**
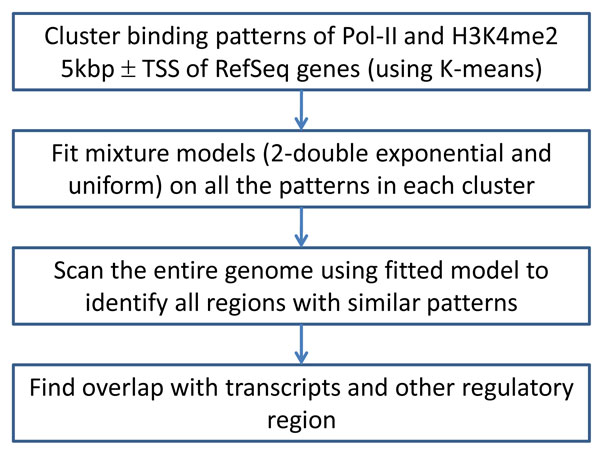
**Analysis Workflow**. Workflow of the analysis done to identify chromatin and histone characteristics of promoters and to find potential novel promoters and other markers.

In the 1*^st ^*step, we characterize Pol-II binding and chromatin mark patterns by performing k-means clustering around the gene transcription starting site (TSS) of known genes. This is then followed in the second step of fitting a double-exponential and uniform mixture model. At the end of the two step procedure the patterns identified will be used to scan the genome to identify putative promoter regions (see Figure 1).

Specifically, we consider the H3K4me2 and Pol-II ChIP-seq profiles along 10-kb regions surrounding well-annotated TSS in known genes using RefSeq database. Each 10-kb (with 5-kb on each side of TSS) regions contains read counts in bins of size 100-bp. In pre-processing step, we smooth the data using a moving average filter replacing each count in each bin with the average of three consecutive bins. Next, in order to prevent interference from neighboring genes, we exclude genes with TSSs within 10-kb of each other. Furthermore, to prevent degenerate clustering, we remove regions with low binding intensities and low variance. Low binding intensities regions are regions with maximum read counts less than 4 among the 100-bp bins over the 10-kb regions. Low variance regions are defined as regions with variance less than 10th percentile over all 10-kb regions. These filtering criteria result in a dataset consisting a total of 9,859 10-kb regions. K-means clustering using correlation as distance measurement is then performed to find sets of common patterns. The optimal number of cluster is determined using *silhouette *values [11]. Larger value of silhouette indicates greater similarity of these patterns within a cluster compared to between clusters. In our application, we found clustering these 9,859 regions into 4 common patterns yields the highest silhouette. Next, we modeled the characteristic signature of Pol-II and H3K4me2 within each cluster using a double-exponential and uniform mixture. The double exponential components will be able to capture both unimodal and bimodal distribution. This is essential because Pol-II and H3K4me2 peaks has been shown to be unimodal and bimodal, respectively. The uniform component will be used to model the tails of Pol-II profiles.

**Figure 2:**
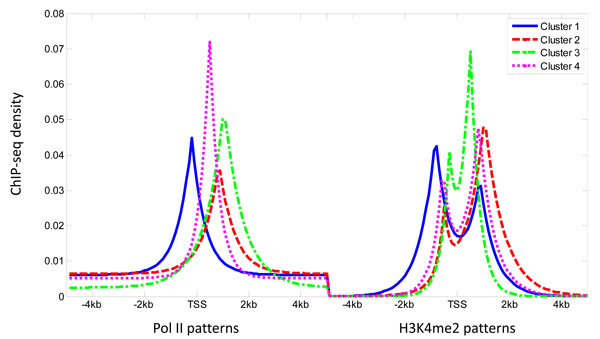
**Fitted Pol II and H3K4me2 patterns**. The 4 distinct profiles of Pol II and H3K4me2 fitted by the double exponential uniform mixture model.

Let *y*_1_(*t*) and *y*_2_(*t*) be the read counts of Pol-II and H3K4me2 ChIP-seq in the 10-kb region around TSS of a gene where *t *is an indicator variables denoting the bin index, respectively. If we quantify the data into bins of size = 100-bp, then *t *∈ *T *= {-50,- 49, ..., 49, 50}. Let *R*(*t*) be the chromosomal region relative to the TSS of the gene. Thus, for *t *= -50, *R*(*t*) denotes region 4901-bp to 5000-bp upstream of TSS. The mixture model for each profile (i.e. Pol-II and H3K4me2) can be defined as follows:

(1) $$f_{k}\left(t\right)=\pi_{1}\frac{e^{-\left|\frac{t-\mu_{1}}{\beta_{1}}\right|}}{2\beta_{1}}+\pi_{2}\frac{e^{-\left|\frac{t-\mu_{2}}{\beta_{2}}\right|}}{2\beta_{2}}+\pi_{3}\frac{1}{b-a}\forall t\in T$$

where *μ *and *β *are the location and scale parameters of the double exponential distribution, respectively. *π *is the mixing proportion (i.e. $\overset{3}{\underset{i=1}{\sum }}\pi_{i}=1$), *a *and *b *are the parameters of the uniform distribution. *k *= 1, 2 for fitting Pol-II and H3K4me2 ChIP-seq profiles respectively. Each model is fitted by minimizing the Kullback-Leibler distance [12] to *f_k_*(*t*) as follows:

(2) $$min\underset{t}{\sum }y_{k}\left(t\right)log\frac{y_{k}\left(t\right)}{f_{k}\left(t\right)}$$

using generalized pattern search (GPS) algorithm [13]. GPS method is a derivatives-free optimization algorithm using positive spanning directions. The GPS algorithm is run until one of the following criteria is satisfied: (1) the number of function evaluations reaches 20,000; (2) maximum number of iterations the algorithm performs reaches 2000; (3) the minimum distance between the current points at two consecutive iteration is less than 10^-6^, (4) After a successful poll, the difference between the function value at the previous best point and the function value at the current best point is less than 10^-6^. The search algorithm is repeated 16 times with different initial points. Using this strategy, we obtained four distinct models of Pol-II and H3K4me2 signatures representing the majority of the patterns exist at promoter region of known genes. Each model is a mixture of double exponential and uniform components. Figure 2 shows the 4 distinct patterns modeled by the finite mixture.

**Figure 3:**
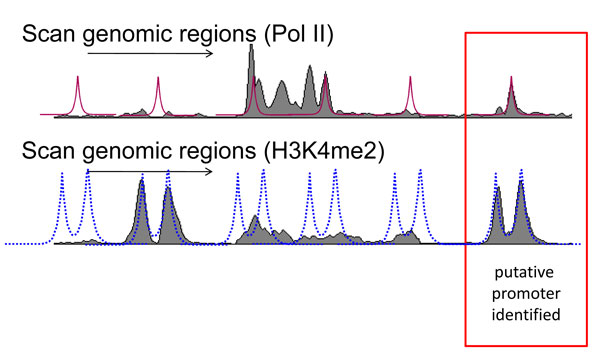
**Schematic of the scanning process to find regions with promoter patterns**. Scan genome using the fitted models to find correlated regions. Gray area (top) is the ChIP-seq data of Pol-II and gray area (bottom) is the ChIP-seq data of H3K4me2. Red curve (top, red-solid-curve) is the double exponential-uniform model fitted to the Pol-II patterns and blue curve (bottom, blue-dot-curve) is the double exponential-uniform model fitted to the H3K4me2 patterns. Correlated regions are identified as region which have significantly high correlation coefficient with both Pol-II and H3K4me2 models (curves).

Finally, we scan the whole genome using the fitted models to find regions that display these Pol-II and H3K4me2 patterns (see Figure 3). We concatenate the fitted Pol-II and H3K4me2 models then use a sliding window of 10-kb moving 1-bp at a time to find regions with the Pol-II and H3K4me2 fitted model. Once genome-wide correlation with these models have been obtained, a threshold for these values must be established in order to classify regions as putative promoters which display these promoter signatures. A null distribution of the test statistics (correlation) are approximated by randomly permuting the read counts of the H3K4me2 and Pol-II regions and calculating their correlation with the fitted model. Regions with high correlation coefficients are defined as regions that have correlation greater than a threshold *z*. The threshold *z *is chosen as the 95*^th ^*percentile of the asymptotic distribution of the test statistics. These genomic locations which display these specific patterns of Pol-II and H3K4me2 are designated as *potential promoters*. For brevity, we will refer to the fitted Pol-II and H3K4me2 patterns as promoter patterns. We further annotate these correlated regions as known promoters and predicted alternative promoters using RNA-seq data in MCF7 along with transcripts databases such as ESTs, snoRNA/miRNA downloaded from USCS genome browser.

## Results

**Figure 4:**
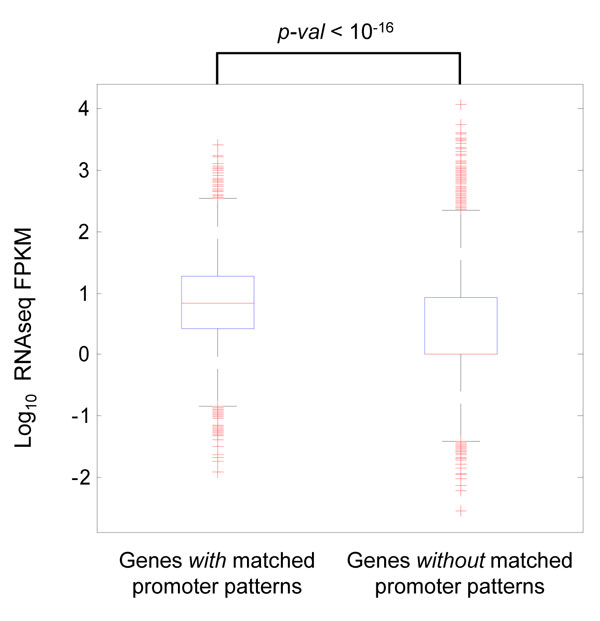
**Genes with promoter patterns have higher expression values**. Genes whose promoter regions display the Pol-II and H3K4me2 patterns have significantly higher expression value than genes which do not have the same promoter patterns (Mann-Whitney test, p-value < 10^-16^).

Scanning the entire genomic region for promoter patterns, we found 7,235 highly correlated regions. These are the regions that show high similarity with any of the four patterns modeled by the double-exponential and uniform mixture models. Around 58% (4167) of these matched regions overlapped with known promoter regions (1-kb upstream and downstream of the RefSeq TSSs). Although these regions only represent 22% of the entire known promoters, it is not surprising as it has been known that not all genes are expressed at the same time. Hence, these promoter patterns may represent those that are currently active in the breast cancer model MCF7 cell line. Indeed as shown in Figure 4, genes whose promoters display these patterns have a significantly higher expression values compared to genes which do not (Mann-Whitney test, p-value *<*10^-16^). Genes expression are determined using FPKM (Fragments Per Kilobase of transcript per Million mapped reads) values derived from RNA-seq data on MCF7 using CuffLink [14].

**Figure 5:**
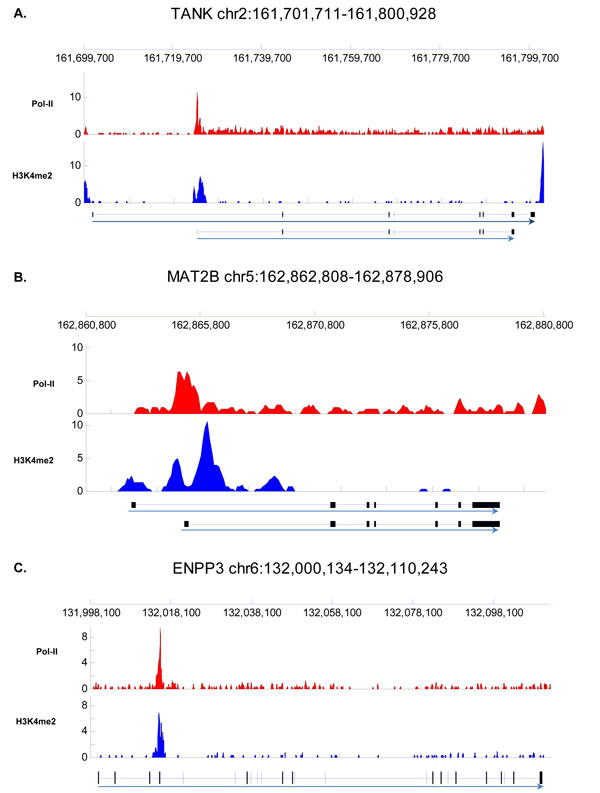
**Promoter patterns are present in the gene bodies**. Exons (black bar) and transcriptional orientation (arrow) are indicated at the bottom of each panel. The location of the longest isoform is indicated at the top of each panel. (A) Promoter pattern exists at the starting site of isoform of *TANK *gene on chromosome 2. (B) Promoter pattern also exists at the starting site of isoform of *MAT2B *gene on chromosome 5. (C) Promoter pattern overlap with exon of gene *ENPP3*.

For the rest of highly correlated regions (3,068) which cannot be mapped to known genes, we found 1,104 of them falls inside known gene bodies. Some of them are known isoforms. For example gene *TANK *on chromosome 2 has been found to have isoforms. Interestingly, as shown in Figure 5A, the transcription starting site for its isoform coincide with the location where the promoter pattern is identified. Alternative promoter of gene *MAT2B *also display the promoter pattern (see Figure 5B). This is evidence of the existence of the promoter pattern in the alternative promoter regions. On the other hand, there are regions showing the promoter pattern which do not overlap with any known isoform. Some of such regions overlap with exons which indicate that these region are very likely be an unknown alternative promoters (see Figure 5C).

**Figure 6:**
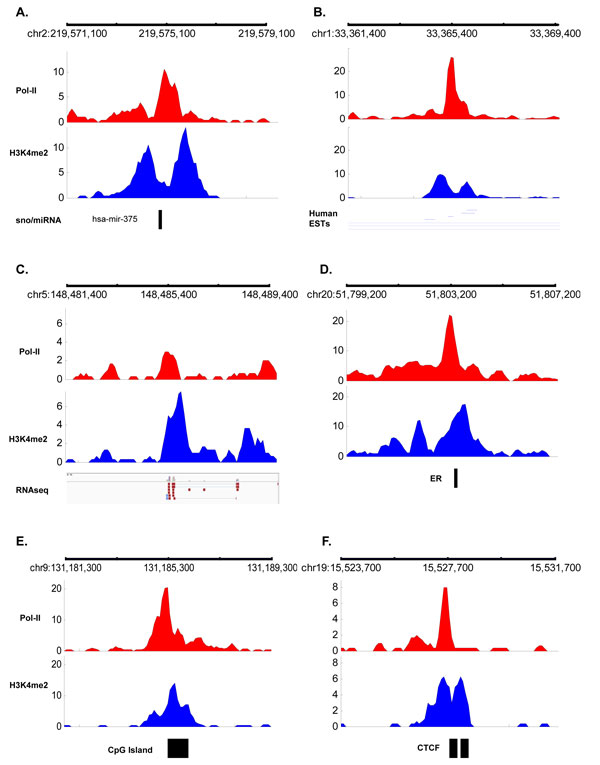
**Regions displaying promoter patterns that overlap with transcripts or other regulatory regions**. (A) Region that overlap with non-coding RNA (hsa-mir-375) on chromosome 2. (B) Region that overlap with 7 human ESTs. (C) Region that is overlap with detected transcript in RNA-seq data. (D) Region that is overlap with ER binding site. Examples of regions displaying promoter patterns. (E) Region that overlap with CpG island (F) Region that overlap with CTCF. Regions are found hierarchically. Hence region that overlap with CTCF do not have an overlap with any other annotation.

For the rest of correlated regions (1,964), we went to find whether these regions can be associated with any transcripts. In order to do this we first find whether there is overlap between these correlated regions and non-coding RNA tracks (i.e. snoRNA and miRNA) from UCSC genome browser as the RNA-seq protocol does not yield data for small RNAs. We found only 6 regions overlap with the location of non-coding RNA in human genome. One example of this region is shown in Figure 6A. Next, we try to find whether the rest of the regions (1,958) have an overlap with human transcripts listed in the expressed sequence tags (EST) database (from UCSC genome browser). The human ESTs are single-read sequences that usually represent fragments of transcribed genes. We found 1,330 regions that overlap with ESTs. An example of this region is shown in Figure 6B. We have also used RNA-seq data on MCF7 to find transcripts of new (undiscovered) genes. RNA-seq data are processed using CuffLinks [14] to assemble transcripts. We found four regions which cannot be mapped to other transcripts but are found to be in the proximity of transcripts detected using RNA-seq data. Example of this region is shown in Figure 6C. Detected transcript image is generated using Integrative Genomics Viewer (IGV) [15]. An overlap with these transcripts is defined as any base pair overlap between the 2-kb area surrounding the center of correlated regions with the starting and end location of the transcripts. A total of 1,340 regions (68%) out of 1,958 region that cannot be mapped to known promoters and their gene body are found to be overlapped with transcripts annotated as non-coding RNAs, ESTs and also those that are detected by RNA-seq. We annotate these 1,340 as predicted alternative promoters as they are shown to be overlapped with some type of transcripts either non-coding or predicted using RNA-seq data.

Recently there has been new discovery on the presence of RNA polymerase II at enhancer regions. These regions which are found to affect genes far away can manufactured their own RNA molecules. Thus, we try to find whether the same promoter pattern can be found at enhancer regions. We used the binding sites of ER (Estrogen Receptor) and AR (Androgen Receptor) as representative of the enhancer regions since both of these protein have been shown to bind at distal enhancer region. Overlapping unmapped region with ER binding sites, we found 120 regions with similar promoter patterns. This region is shown in Figure 6D. However, after mapping ER binding sites, we did not find any overlap with AR binding sites.

**Figure 7:**
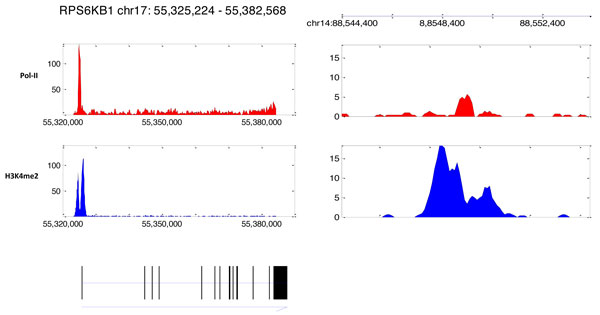
**Example of regions with Pol-II (top-red) and H3K4me2 (bottom-blue) patterns**. **Left panel**, the region predicted has an overlap with a RefSeq gene called *RPS6KB1*. Transcriptional orientation (arrow) is indicated at the bottom. **Right panel**, a potential promoter is predicted at chromosome 14 at around 88,549,400. However no overlap is found with neither RefSeq, transcripts or other regulatory regions.

**Figure 8:**
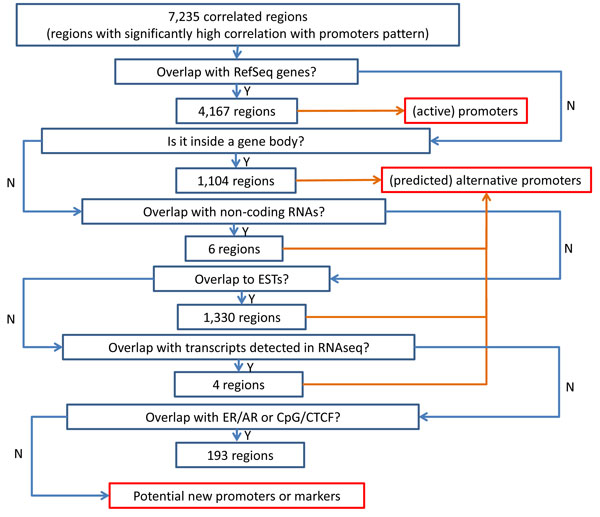
**Summary of overlap of correlated regions with genome annotation, transcripts and other regulatory regions**. Number of regions displaying promoter patterns that are found to be overlapping with genome annotation, transcripts or other regulatory regions. The search for regions that overlap was done hierarchically. Hence at the end, the unmapped regions are region that do no overlap with any of the genome annotation, transcripts or other regulatory regions.

We found 73 out of the rest of the correlated region (504) can be further mapped to other regulatory regions such as CpG island and CCCTC binding factor (CTCF). We used CpG island tracks downloaded from UCSC genome browser to annotate CpG island location. For CTCF, we used the CTCF binding sites that are present in three different cell lines (Jurkat, CD4 and HeLa) since it has been shown that these sites are conserved [16]. Example of regions mapped to CpG island and CTCF binding sites are shown in Figure 6E and 6F, respectively. Finally, we ended up with 431 region that display the promoter pattern which cannot be mapped to neither known genes, transcripts nor any regulatory regions. Example of this region is shown in Figure 7 (right panel). Ultimately, these unmapped regions may very much be potential new promoters or markers for other annotation that needs further investigation. Figure 8 shows the summary of the overlaps which are done hierarchically from top to bottom. The number of regions that independently matched to each genome annotation is summarized on Table 1.

**Table 1:** Number of correlated regions that overlap with each genome annotation or transcripts including those that are detected using RNA-seq

Annotations	Number of overlaps
RefSeq	4167
Gene body	1177
ncRNAs	21
ESTs	6586
RNA-seq	2707
ER	800
AR	224
CpG island	4838
CTCF	519

**Figure 9:**
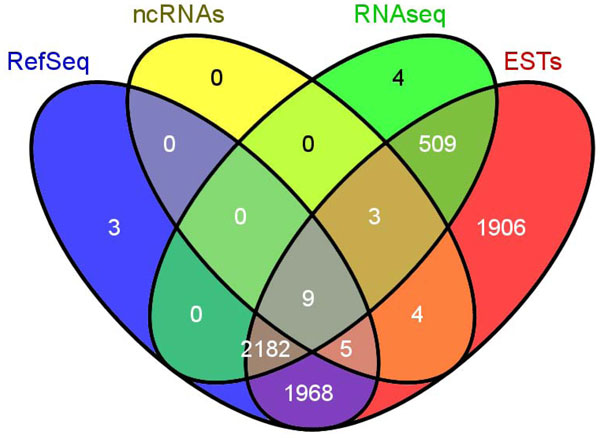
**Venn diagram of correlated region that overlap with more than one genome annotation**. Most of the region that display promoter patterns overlap with RefSeq genes, ESTs and RNA-seq (2182 regions).

We investigated the overlap of these correlated regions with more than one genome annotation (Figure 9, image is generated using Venny [17]). We found that almost all of the correlated regions that overlap with RNA transcripts also overlap with EST (99%,2703 out of 2707). There are about 26% of correlated regions which exclusively map to ESTs and only 3 map exclusively to TSS of RefSeq genes. There are still about 5% (431) of the correlated that do not overlap with known genes, transcripts or other regulatory regions, they may still represent potential novel promoters. For example, Figure 7 (left panel) shows an example of a putative promoter region that overlap with a known gene called *RPS6KB1 *on chromosome 17. The Pol-II and H3K4me2 patterns are very prominent around the TSS of this gene with the combination of unimodal Pol-II peak and the bimodal H3K4me2 peak. Figure 7 (right panel) shows an example of a putative novel promoter region that does not overlap with any of the above genome annotations. Although, the pattern on the right also display unimodal Pol-II peak and bimodal H3K4me2 peak just like the known promoter pattern on the left, it does not have tails in the transcribed region. As we have discussed earlier, this phenomenon could be due to Pol-II stalling [7].

## Discussion

In this paper, we develop a novel algorithm based on finite mixture model to predict promoter regions using ChIP-seq profiles. We are interested in identifying transcriptionally active promoters clustering all TSSs regions. We use the term promoter to describe these regions throughout the paper. We identified putative promoter regions based on their statistical significance. Our algorithm takes advantage of the new sequencing technology which allow one to observe the binding patterns by modeling the shape of these promoter patterns instead of simply categorizing binding sites as binary (present/absence) [18]. Four common models representing shapes of promoter patterns are obtained by K-means clustering algorithm. Although these patterns appear to be similar, the shift in the location of peaks may be meaningful. For example, the shift may indicate genes that are poised to be transcribed but not yet active. Furthermore, the distinctive patterns may prove to be important in differentiating different functions or different behavior of these promoters. More detailed investigation is needed in order to draw more clear picture of the gene expression mechanism. Nevertheless, the proposed algorithm may help with the discovery of novel promoters (including alternative promoters) and aid in the ongoing annotation of promoters from different ChIP-seq experiments. Finally, the proposed algorithm may also be extended to identify enhancers elements important in distal gene regulation. For instance, in 6C, the combined Pol-II and H3K4me2 peaks mapped to a potential enhancer region with detectable transcripts in the RNA-seq experiment. These short transcripts are likely to be the recently discovered eRNA which are short RNA transcribed from enhancer regions even though its function is still not clear [19]. These findings will lead to new insight on the epigenetic mechanisms on transcription regulation with applications in cancers.

## Competing interests

The authors declare that they have no competing interests.

## Authors' contributions

CT collected datasets, performed all the data analyses and drafted the manuscript. SL, KH and CT designed the study and wrote the manuscript. SL, KH and THMH conceived the study and directed the whole research work. THMH provided the ChIP-seq data. All authors read and approved the manuscript. Correspondence and requests for materials should be addressed to CT (taslim.2@osu.edu), KH (kun.huang@osumc.edu) or SL (shili@stat.osu.edu).
